# Using publicly visible social media to build detailed forecasts of civil unrest

**DOI:** 10.1186/s13388-014-0004-6

**Published:** 2014-09-03

**Authors:** Ryan Compton, Craig Lee, Jiejun Xu, Luis Artieda-Moncada, Tsai-Ching Lu, Lalindra De Silva, Michael Macy

**Affiliations:** Information and System Sciences Laboratory, HRL Laboratories, 3011 Malibu Canyon Road, Malibu, 90265 CA USA; Department of Computer Science, University of Utah, Salt Lake City, Utah USA; Social Dynamics Laboratory, Cornell University, Ithaca, New York USA

**Keywords:** Information retrieval, Data and text mining, Computational social science

## Abstract

We demonstrate how one can generate predictions for several thousand incidents of Latin American civil unrest, often many days in advance, by surfacing informative public posts available on Twitter and Tumblr.

The data mining system presented here runs daily and requires no manual intervention. Identification of informative posts is accomplished by applying multiple textual and geographic filters to a high-volume data feed consisting of tens of millions of posts per day which have been flagged as public by their authors. Predictions are built by annotating the filtered posts, typically a few dozen per day, with demographic, spatial, and temporal information.

Key to our textual filters is the fact that social media posts are necessarily short, making it possible to easily infer topic by simply searching for comentions of typically unrelated terms within the same post (e.g. a future date comentioned with an unrest keyword). Additional textual filters then proceed by applying a logistic regression classifier trained to recognize accounts belonging to organizations who are likely to announce civil unrest.

Geographic filtering is accomplished despite sparsely available GPS information and without relying on sophisticated natural language processing. A geocoding technique which infers non-GPS-known user locations via the locations of their GPS-known friends provides us with location estimates for 91,984,163 Twitter users at a median error of 6.65km. We show that announcements of upcoming events tend to localize within a small geographic region, allowing us to forecast event locations which are not explicitly mentioned in text.

We annotate our forecasts with demographic information by searching the collected posts for demographic specific keywords generated by hand as well as with the aid of DBpedia.

Our system has been in production since December 2012 and, at the time of this writing, has produced 4,771 distinct forecasts for events across ten Latin American nations. Manual examination of 2,859 posts surfaced by our method revealed that only 108 were discussing topics unrelated to civil unrest. Examination of 2,596 forecasts generated between 2013-07-01 and 2013-11-30 found 1,192 (45.9*%*) matched exactly the date and within a 100 km radius of a civil unrest event reported in traditional news media.

**Electronic supplementary material:**

The online version of this article (doi:10.1186/s13388-014-0004-6) contains supplementary material, which is available to authorized users.

## Introduction

Widespread adoption of social media has made it possible for any individual to rapidly communicate with an audience of thousands [1]. Unlike traditional news media, where several difficult time-consuming steps must be carried out prior to publication and the possibility of censorship by media owners is ever-present, information in social media becomes publicly available within a few seconds of its creation and often circumvents attempts at content filtering.

Recently, the speed and flexibility of publication on social media have motivated its use as a tool for the organization and announcement of strikes, protests, marches and other demonstrations to the public (hereinafter collectively referred to as “civil unrest”) [2]. In this work, we show in detail how it is now possible to examine social media and report on a large number of civil unrest events prior their occurrence, while they are still in their planning stages. We restrict our attention to publicly visible data only. In fact, we restrict our analyses only to data that has been explicitly flagged as public by its creator. Information such as IP addresses (which can be used for geolocation) or connection speed (which may correlate with large protests [3]) is ignored in this study.

Early detection of civil unrest events is valuable for several industrial and government applications. For example, if a port is likely to shut down due to a riot, shipping companies may opt to redirect freight in order to prevent unexpected losses. If a massive protest is planned to happen in front of an embassy, governments may elect to postpone diplomatic visits in order to ensure the safety of their politicians. The value of civil unrest forecasting has recently caught the attention of researchers from a wide variety of disciplines [4-7].

Predicting international protests by mining Twitter for mentions of future dates was first done in [8] (which this work is an extension of). Later research by Kallus [9] adapted the future date heuristic to forecast unrest in additional languages and developed a new evaluation methodology. Research by Xu et al. in [10] demonstrated results focused specifically on Tumblr.

Alternative methods for civil unrest forecasting are based on physical models describing large-scale theories of population behavior (e.g. [6,7,11]). Often relying on time series (or “trends”), these methods take into account a small amount of information from millions of posts, treating as social media as a sensor of population sentiment. While time series analysis may lead insight into collective social dynamics, relying on millions of tweets to generate predictions for the next day’s events is not practical when the number of events is high and detailed information from each forecast is important. Time series based methods suffer a major disadvantage when an auditor seeks additional information about a given prediction. Expecting all auditors to fully grasp the models employed to generate the prediction is unreasonable; having the auditors examine all posts that were used to generate the time series is impossible.

The distinguishing feature of our approach is direct extraction and analysis of a small number of highly relevant posts, treating social media as a “news source” rather than a “sensor”. This allows us to easily generate a large number of predictions each day and allows an auditor to easily read through all the posts associated to each prediction.

The data input to our system consists of all public posts on Twitter and Tumblr. Our decision to work with Twitter and Tumblr and not, say, Facebook, Google+, LinkedIn, or Orkut, is primarily motivated by the fact that high-volume data feeds consisting of public posts on Twitter and Tumblr are readily available from several data providers [12,13]. Additionally, Twitter has recently gained much notoriety as an organizational tool for activism after its central role in 2011 Arab Spring protests [14,15]. Tumblr, however, has not yet been the focus of much research and little is known about its structure or utility. We will show that, while the number of forecasts we generate with Tumblr is eclipsed by Twitter, much information about future civil unrest is in fact present and easily retrievable from Tumblr.

The focus of our work is Latin America. Widespread use of Twitter and Tumblr, numerous strikes and protests, absence of government censorship, and only two languages throughout the region make this an ideal location to study social media signal prior to civil unrest events. Our research is distributed across ten major nations: Argentina, Brazil, Chile, Colombia, Ecuador, El Salvador, Mexico, Paraguay, Uruguay, and Venezuela.

This paper is organized as follows: section ‘Method’ describes each step of our technique in detail. Section ‘Results’ showcases our user interface and has information about the system’s past performance. Finally, section ‘Conclusion’ discusses future work and concluding remarks.

## Method

Our goal is to generate forecasts of the form: 

Where “population” describes the demographic of the event participants (eg education, labor, agriculture), “event_type” gives further detail about the reason for the event (eg employment, housing, economic policies), “date” is the date the event is forecast to occur on, “location” is the city where we expect the event to occur, and “probability” is how likely it is that the event will actually happen.

We extract informative social media posts via the application of several filters (cf Alg. 1) designed to reduce the number of posts we analyse down from hundreds of millions to dozens. The posts identified by alg. 1 are often rich in information about upcoming civil unrest. We believe that a single human auditor could easily read all posts in *t*_5_ for a given day and be well-informed about several announced events. In the following subsections we describe the filters to reach *t*_5_ in detail. 

### Keyword searches

The first filter a tweet must pass is a simple check for mentions of Latin American civil unrest keywords. We have manually identified a collection of 44 keywords which we believe are highly relevant to civil unrest (e.g. “protesta”, “huelga”, “marcha”). The advantage of this filter is that it is possible to apply it to the entirety of Twitter and Tumblr with minimal effort.

### Future date searches

Simple checks for keyword mentions are poor indicators of content. A quick experiment has shown that, in both English and Spanish, only about 20% of posts that contain a civil unrest keyword are indeed about civil unrest. Furthermore, it is unclear how to forecast an event date from only posts with certain keywords. We thus apply a second filter, one for mentions of future dates, to the posts containing unrest keywords.

Our temporal expression tagger searches first for month names and abbreviations in Spanish and Portuguese and second for numbers less than 31 within three whitespace separated tokens from each other. Thus, an example matching date pattern would be “10 de enero”. Four-digit years are rare in tweets, in order to determine the year of the mentioned date we use the year which minimizes the number of days between the mentioned date and the tweet’s post time. In our example, if a tweet mentions “10 de enero” on 2012-12-29 we assume the user is talking about 2013-01-10 as 2013-01-10 is closer in time to 2012-12-29 than 2012-01-10 is. Additionally, we tag colloquial date expressions (e.g. “el martes próximo”) with basic string searches. Despite the simplicity of this approach, we find that many posts can be annotated with our date tagger. More advanced temporal expression taggers, such as Heideltime [16] may be used in place of our method for Spanish text, but are currently not available in Portuguese.

Once we have extracted dates from the text, we assert that the mentioned dates occur after the tweets post time.

When the future date filter is applied the number of tweets is reduced substantially, a quick experiment on 144,167 tweets containing unrest keywords collected on 2013-03-01 found that only 1,512 of these tweets also contained future dates.

Social media text is remarkably short. On Twitter there is a hard limit of 140 characters per tweet, and Tumblr posts (which are primarily focused on images) rarely exceed the length of tweets. When an unrest keyword is mentioned alongside a future date there is little room left to obscure the topic of the post away from civil unrest. We find this comention filter to be highly informative.

For each tweet passing this filter we tentatively issue a forecast for the mentioned date.

### Logistic regression classification

Comentions of keywords with future dates, however, does not guarantee that a particular post is indeed about civil unrest. For Twitter, we have developed two classifiers to classify tweets based on their relevance to a civil unrest event. Our first classifier is a standard logistic regression classifier trained on tweets. The features for the classifier were unigrams and bigrams that surpassed a frequency threshold of 3 in the training data. The training data was acquired using three annotators through Amazon Mechanical Turk and they annotated 3000 tweets for their relevance to a civil unrest event (pairwise inter-annotator agreement ranged from 0.68 to 0.74).

Our second classifier makes use of recent work we have done establishing that tweets from organizations are roughly three-times more likely to be civil unrest-related than similar tweets from individuals [17]. In order to exploit this concept, we designed an auxiliary classifier that classifies the source user type of a tweet into two categories - organizations and individuals. For this classifier, we make use of an ensemble framework for user type identification based on heuristics, an *n-gram* classifier, and a linguistic classifier. The heuristics were designed to capture two strong cues that are characteristic of organization tweets - 1) they almost always contains a URL and 2) organizational tweets rarely contain replied tweets (tweets beginning with @user mentions). The *n-gram* classifier was based on unigrams and bigrams and the linguistic classifier captures several types of linguistic features that are characteristic of tweets in either category. These three components in the ensemble are then utilized in linear combination using another logistic regression classifier to determine the user type of any given tweet. After we have identified the posting user as individual/organization using this classifier, we adjust the forecast probability accordingly, by incorporating the likelihoods to derive the posterior probability of a tweet being civil unrest-related given its user type.

### Event geocoding

Identification of event locations is central to the goal of this project. We infer the location of an upcoming event with two different methods, one text based and the other social network based.

Our text based location assignment is a straightforward search for mentions of cities or monuments from a manually compiled list of unambiguous location names. For Tumblr, where GPS information is never public, event geocoding is solely textual. For Twitter, where GPS information is public, but extremely rare, we are able to use social network based techniques to infer additional user locations (see 3 for detail on our user geocoder).

For each tweet passing the logistic regression filter, we identify user IDs of all the tweet’s retweeters. User IDs are then fed into our user geocoder and filtered based on whether or not they center in Latin America. We assign a latitude and longitude to the forecast event using a robust estimate of the center of the retweeter’s locations, i.e. the forecast location is the *l*1-multivariate median [18] of the retweeter locations.

To be precise, let  be the set of all retweeter locations and *d* the geodesic distance measured using Vincenty’s formulae [19]. We compute the *l*1-multivariate median of  as:

(1) $$  \underset{x}{\arg\!\min} \; \sum_{y \in \mathcal{U}} d(x,y)  $$

and use the solution to eq. 1 for event location.

The success of our geocoding depends on communicative locality in Twitter, which is currently an unsettled research direction. Work supporting the idea that social ties in Twitter are grounded in geography can be found in [20-23]. Similar work on the Facebook social network was done in [24]. These papers study communicative locality by restricting attention to subsets of the social networks where all users locations are known. Results of [20] demonstrate that @mentions are unlikely to align with geography unless the @mentions have been reciprocated.

Research showing that Twitter contact is not grounded in geography can be found in [25], where the author examines a 32.5 million GPS-known retweet pairings and finds an average distance of 749 miles between users. Averages, however, are sensitive to outliers which may be present in the social media data studied. In this work, we will make use of robust statistics (i.e. the *l*1-multivariate median and median absolute deviation) to estimate center and spread for sets of locations.

The papers mentioned thus far have been focused on user geocoding and do not guarantee that event location can be learned from social media sharing patterns. Predicting an event location from retweeter location is only possible for tweets containing event announcements (which are the focus of this work).

We quantify the dispersion of a set locations using the median absolute deviation,

(2) $$  \overset{\sim}{\mathcal{U}} = \text{median}_{i} \left(d(u_{i}, \; l1\text{-median}(u_{j})) \right)  $$

**Figure 1:**
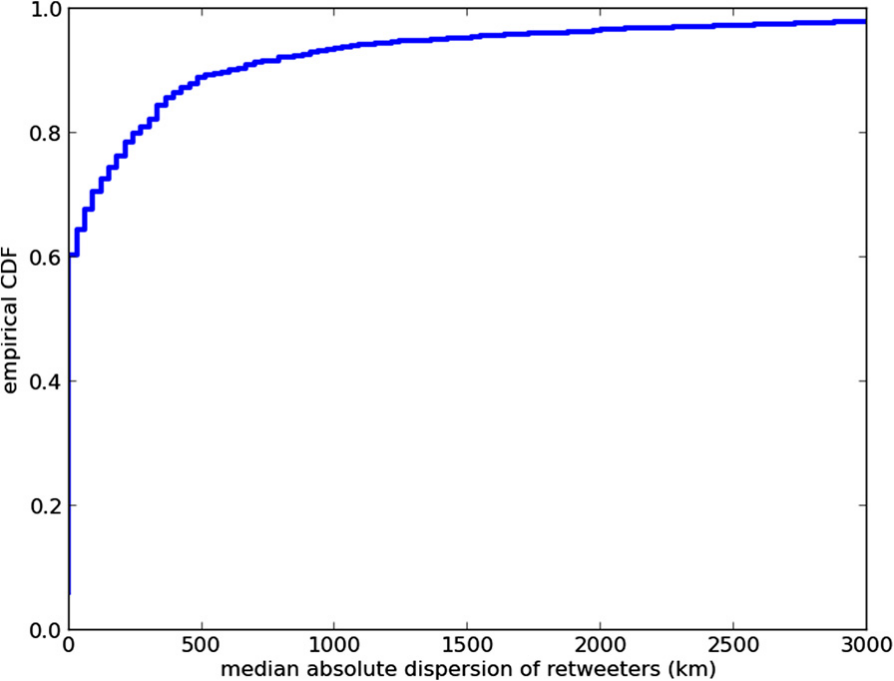
**Empirical CDF of the median absolute deviation of retweeter locations of 4,004 forecasts generated by our model.** With over 80*%* probability the retweeters are dispersed by less than 500 km.

Examining 4,004 forecasts generated by our model with more than 3 geocodable retweeters shows us that the vast majority of tweets used to generate forecasts have localized retweeters. In Figure 1 we show the empirical CDF of the median absolute deviation of retweeter locations, the data indicates that there is over an 80*%* probability the retweeters are dispersed by less than 500 km. While the 500 km figure may be too large to disentangle neighbouring cities, the number of tweets surfaced by our method is small enough that a human could manually read through them before any action is taken.

**Figure 2:**
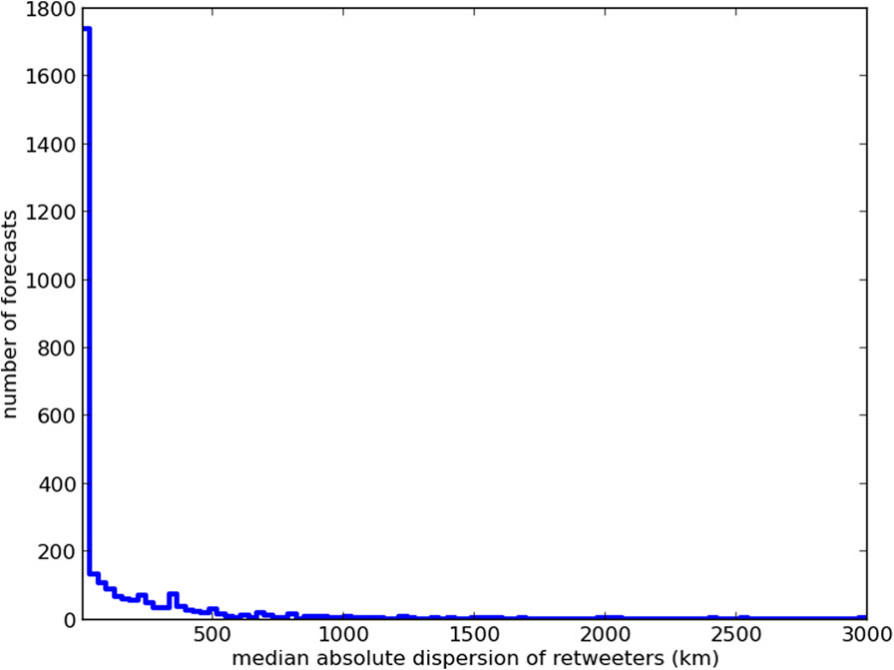
**Histogram of forecasts per retweeter dispersion level.** Retweeters typically localize within a small radius. We take the center of the retweeter locations to be the forecast location.

**Figure 3:**
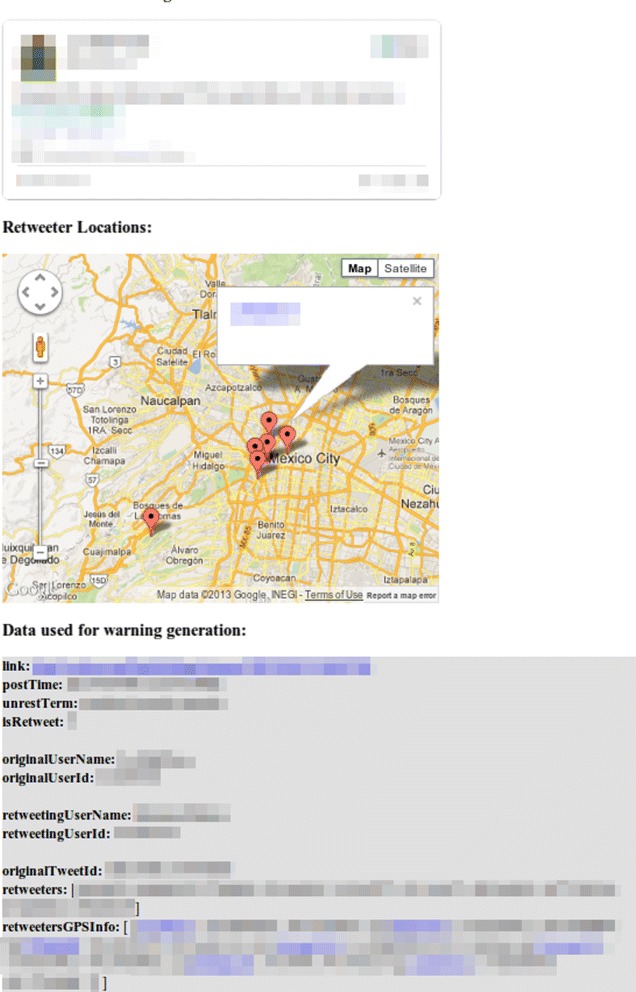
**Example forecast.** A march related to Petroleos Mexicanos (Pemex) is planned for March 18 in Mexico City. Our system detected the event on March 5th. The interactive map provides end-users with links to retweeter accounts.

In Figure 2 we examine the same 4,004 forecasts and plot the number of forecasts at each dispersion level, the histogram shows that a large number of forecasts have retweeters localizing within a small radius. An example of this phenomenon is visible in Figure 3, where we see that all retweeters discussing the upcoming march are localized within Mexico City.

We note here that this filter is remarkably difficult to pass. Of the 1,512 tweets collected in the previous step in our example, only 36 passed the geocoding filter.

We also remark that, unlike much research in social media analysis, our event geocoding technique is entirely language independent. Which opens up the possibility of expanding our method to a global scale.

#### User geocoding

We identify retweeter locations with our previously developed Twitter geocoder [20,23]. In this section we briefly explain how our geocoder works, more detail is available in [20] and [23].

The distinguishing feature of our geocoder is that we iteratively infer a non-GPS user’s location based on the locations of their friends. This is accomplished by solving the convex optimization:

(3) $$  \min_{\mathbf{f}} | \nabla \mathbf{f} | \; \text{subject to} \; f_{i} = l_{i} \; \text{for} \; i \in L \; \text{and} \; \overset{\sim}{\nabla f_{i}} \leq \gamma  $$

where *f* encodes a location estimate for each user, *L* denotes the set of users who opt to make their GPS locations, *l*_*i*_, public, the total variation, |∇**f**|, on the Twitter @mention network is defined by:

(4) $$  | \nabla \mathbf{f} | = \sum_{ij} w_{ij} d\left(\,f_{i},f_{j}\right)  $$

Here, the edge weights, *w*_*ij*_, are equal to the minimal number of reciprocated @mentions between users *i* and *j*. The quantity $\overset {\sim }{\nabla f_{i}}$ is the median absolute deviation of the users distances to their friends, defined by

(5) $$  \overset{\sim}{\nabla f_{i}} = \text{median}_{j} \left(w_{ij}d\left(\,f_{i},f_{j}\right) \right)  $$

The parameter *γ* defines how dispersed we allow a user’s friends to be and is set to 100 km in our code.

In summary, we seek a network such that the sum over all geographic distances between connected users is as small as possible, subject to a constraint on the dispersion of each user’s friends.

Our geocoding technique falls under the category of transductive learning and shares some similarity with “label propagation” [26]. However, unlike label propagation, our labels (latitude/longitude pairs) are continuously valued. Equation 3 exploits this additional structure with geodesic distance and total variation, which has demonstrated superior performance as an optimization heuristic for several information inference problems across a wide variety of fields [27-29].

We begin by extracting home locations for users based on the number of times they have tweeted with public GPS. When we observe 3 or more tweets from a user within a 30 km radius we use the geometric median of those tagged tweets to establish the user’s home location. This provides us with home locations for 10,590,474 users. We extract self-reported locations when a users enters an unambiguous location name into their profile. The number of users we find from self-reports is 9,466,251, of these 8,057,879 were not using GPS publicly. We hold out 10*%* of GPS users for testing. By combining self-reports with non-test GPS users we obtain locations for 17,589,170 Twitter users. These 17M users are used for *L* in eq. 3.

The total variation functional is nondifferentiable. Solving a total variation-based optimization is thus a formidable challenge and vastly different methods have been proposed for several decades [30].

We employ “parallel coordinate descent” to solve eq. 3. Most variants of coordinate descent cycle through the domain sequentially, updating each variable and communicating back the result before the next variable can update. The scale of the data we work with necessitates a parallel approach, prohibiting us from making all the communication steps required by a traditional coordinate descent method.

At each iteration, our algorithm simultaneously updates each user’s location with the *l*1-multivariate median of their friend’s locations. Only after all updates are complete do we communicate our results over the network.

Note that the argument that minimizes |∇_*i*_(**f**^*k*^,*f*)| is the *l*1-multivariate median of the locations of the neighbours of node *i*. Thus, we iteratively update each user’s location with the median of their friends locations, provided that their friends are not too dispersed.

We have no convergence proof for Alg. 2. Empirically, Alg. 2 converges, providing us with estimates of home locations for 91,984,163 Twitter users. Comparison with the 10*%* hold-out GPS users shows a median error of 6.65 km, and a mean error of 300.06 km with a standard deviation of 1,131.83 km. 

### Demographics and event code assignment

We condense duplicate forecasts for the same date/location into one forecast by averaging their probabilities.

Language experts have provided us with lists of terms relevant to several demographics and event types in Latin America. Additionally, we greatly expand our lists using DBpedia. As an example, entering the below query into http://dbpedia.org/snorql/?query= will provide a list of all political parties in Argentina or Venezuela. 

Entering the following query will provide a list of all universities in Argentina or Venezuela. 

The two above queries provide us with keywords allowing us to distinguish between politics and education.

To assign a demographic to each forecast we collect the tweet histories of every retweeter of every tweet associated with a forecast and search our lists of terms. The most commonly occurring classes of terms are used to assign our forecast’s demographic and event code.

## Results

Successful end-user interpretation is important. By approaching this problem from the viewpoint of data mining rather than time series analysis we can provide an easily interpretable audit trail with minimal effort. For each forecast generated we provide the tweets used, the retweeter locations, the keywords matched, and links to all retweeter accounts. (cf Figure 3).

**Figure 4:**
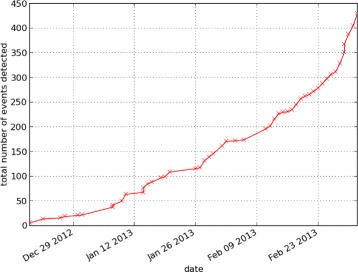
**Cumulative sum of the number of forecasts generated since 2012-12-17.** The increased number of warnings per day in November 2013 was due primarily to improvements in date tagging.

Our system has been in place since 2012-12-17 (cf Figure 4, Table 1, and Table 2), in that time the rate at which forecasts are generated has been steadily increasing as we continue to improve our algorithms and keyword lists.

**Table 1:** **Number of forecasts generated for each country**

**Number of events forecast**	**Nation**
500	Argentina
778	Brazil
317	Chile
557	Colombia
134	Ecuador
69	El Salvador
1235	Mexico
128	Paraguay
65	Uruguay
985	Venezuela

Mexico is highly active on twitter.com and receives the most coverage from our system. Timeframe: 2012-12-17 until 2014-01-14.

**Table 2:** **Total number of forecasts generated by our system**

**Data feed**	**Number of forecasts**	**Average lead time**
Twitter only	5150	3.91
Tumblr only	198	6.38
Both	1298	2.93
Total	6596	3.81

Timeframe: 2012-12-17 until 2014-03-10.

Assessing the performance of our system is relatively straightforward given the audit trails. Manual examination of 2,859 posts surfaced by our method revealed only 108 that were discussing topics related to sporting events, concerts, other public functions, or simple chatter.

It is possible to evaluate such a system without the use of our audit trail. Manually searching major news media for articles describing Latin American civil unrest provided us a ground truth dataset of 4,825 articles describing distinct events between 2013-07-01 and 2013-11-30. In this time frame we generated 2,596 forecasts. We align forecasts with news articles when the forecast date matches exactly with the event date and the forecast location is within 100 km of the event location. We find that 1,192 forecasts could be aligned in this way. A complete description of the manually annotated data used for evaluation can be found in [31].

A completely automated evaluation is possible with the aid of the GDELT dataset [32]. Briefly, the GDELT project aims to automatically extract and annotate all English news articles describing societal-scale events. GDELT uses the CAMEO coding system, where code “14” can be taken to mean civil unrest. Of our 2,596 forecasts, only 583 aligned with GDELT events within 100 km and on the exact date. It is not yet clear why the precision is lower here. Possibilities may be due to differences in publishing criteria between Latin American social media and English traditional news media, geocoding and date tagging inaccuracies, or the fact that our keyword lists are generated without taking into account CAMEO coding. We hope to improve precision on GDELT in future work.

**Figure 5:**
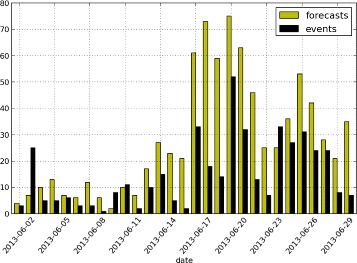
**Number of events forecast to happen per day in Brazil during June 2013.** Our system under reported the initial wave of protests, but successfully captured a major uptick in late June. Average lead time: 5.58 days.

We examine forecasts generated by our method in June 2013. This time period encompasses the “Brazilan Spring” where massive protests swept across the nation. The number of real events per day as well as the number of events forecast to happen per day is shown in Figure 5. These protests generated substantial signal in Twitter. In Brazil, our geocoder reported over 2M tweets from Brazil containing the Portuguese term “protesto” during the month with a peak of over 400,000 per day in late June. A visualization of these tweets is available in Additional file 1.

### Tumblr results

**Figure 6:**
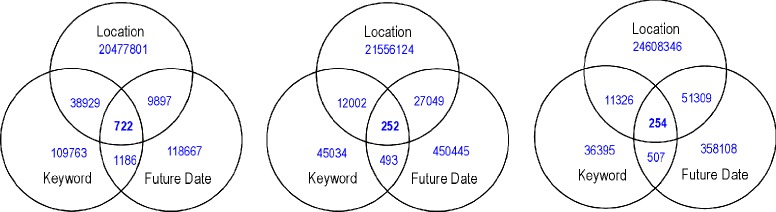
**Venn diagram showing the number of Tumblr posts passing each filter.**

**Figure 7:**
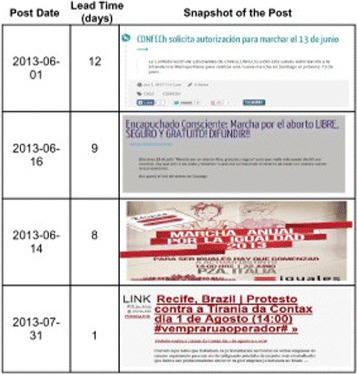
**Snapshots of Tumblr posts (detected by our system) showing planned future civil unrest events.**

Recall that our system consists of a set of filters. The Venn diagrams in Figure 6 show the numbers of resulting Tumblr posts which pass each filter. The number of posts is substantially smaller and more manageable when compared with the original size of input data. The surfaced posts are easy to read and highly informative, cf. Figure 7.

Forecasts from Tumblr and Twitter are fused together when they are forecast to occur on the same date and within the same city. After fusion, we find that roughly 12.7*%* of our forecasts are visible in both Twitter and Tumblr (cf. Table 2). Interestingly, when restricting to June 2013 there is minimal overlap between Twitter and Tumblr (cf Table 3).

**Table 3:** **Number of forecasts generated for June 2013 from the different data feeds**

**Data feed**	**Number of forecasts**	**Average lead time**
Twitter only	525	5.57
Tumblr only	51	5.98
Both	4	2.75
Total	580	5.58

Surprisingly, of the 580 forecasts, only 4 were visible in both Twitter and Tumblr.

In the same manner as before, we evaluate matches against news articles during the 2013-07-01 until 2013-11-30 period. There were 138 warnings based only on Tumblr during this period, 56 (40.5*%*) could be aligned with a manually annotated news articles while 32 (23.1*%*) matched GDELT. There were 11 warnings based on both Twitter and Tumblr, 7 (63.6*%*) of these matched manually annotated news while 3 (27.2*%*) matched GDELT.

## Conclusion

Social media has become a powerful tool for the organization of mass gatherings of all types. However, the shear volume of Twitter and Tumblr make it difficult to automatically identify new and valuable information in a reasonable amount time. In this work we have provided a straightforward approach for the detection of upcoming civil unrest events in Latin America based on successive textual and geographic filters.

Traditional news media is often assumed to be perfectly accurate and can therefore only report on events once they have occurred. The fact that it is now possible to relax the assumption of perfect accuracy and report on events before their occurrence is remarkable and continued work on this project is already in progress.

Immediate future work includes more advanced tweet classification using larger training sets, associated user IDs, our @mention network, and dictionary-based approaches. We also plan to analyse the links shared in tweets for further information on upcoming events.

## Additional file

Additional file 1 Visualization of 2M tweets containing the term “protesto” during June 2013.
